# Complete Heart Block in Young Patient With Systemic Lupus Erythematosus: A Case Report

**DOI:** 10.7759/cureus.117091

**Published:** 2026-09-28

**Authors:** Sorkhan Sok, Lalin Meth, Chanthon Phum, Sophearath Nhem, Phang Sok, Sokha Chan, Chour Sok

**Affiliations:** 1 Department of Cardiology, Calmette Hospital, Phnom Penh, KHM; 2 Department of General Medicine A, Calmette Hospital, Phnom Penh, KHM

**Keywords:** autoimmune disease, case report, complete heart block, myopericarditis, systemic lupus erythematosus

## Abstract

Complete heart block is a rare but serious cardiac complication of systemic lupus erythematosus. Most reported cases present with symptoms such as syncope. In this report, we describe the case of a 33-year-old woman with systemic lupus erythematosus who was diagnosed with asymptomatic Complete heart block. This case highlights the importance of proactive cardiac evaluation, including a baseline electrocardiogram, in systemic lupus erythematosus patients to detect clinically silent conduction defects that may carry a risk of sudden cardiac death.

## Introduction

Systemic lupus erythematosus (SLE) is a systemic autoimmune disorder capable of affecting virtually any organ system; however, while pericarditis represents its most frequent cardiac manifestation, conduction disturbances such as atrioventricular (AV) block are less common, with complete heart block (CHB) remaining a rare and frequently underappreciated complication in the adult population. While syncope typically serves as the most frequent presenting symptom due to severe bradycardia or asystole, asymptomatic CHB is even less common, presenting significant challenges for both timely diagnosis and clinical management [1].

This report describes a case of incidentally identified asymptomatic CHB in a young woman with active, multi-organ SLE, highlighting the importance of maintaining a high clinical suspicion and performing cardiac evaluation in this patient group.

## Case presentation

A 33‑year‑old previously healthy female presented with a two‑week history of daily fever (maximum 38°C), fatigue, generalized myalgia, polyarthralgia (wrists, metacarpophalangeal joints, and knees) and a facial rash that had developed over five days. The patient also noted painless oral ulcerations and reported unintentional weight loss of 4 kg. There was no history of chest pain, palpitations, syncope, pre‑syncope, or dyspnea on exertion. She was not taking any regular medication, had no exposure to toxins, and had no known cardiac disease or family history of sudden death or conduction disorders. On admission, she was febrile (38°C) but hemodynamically stable with a blood pressure of 115/79 mmHg, resting bradycardia at 46 beats per minute (bpm), and an oxygen saturation of 98% on room air. Physical examination revealed a typical malar (“butterfly”) rash with erythematous, slightly edematous plaques over both cheeks and the bridge of the nose, sparing the nasolabial folds. Multiple small painless ulcers were present on the buccal mucosa, along with oral mucosal petechiae and palmar erythema with purpura (Figure 1).

**Figure 1:**
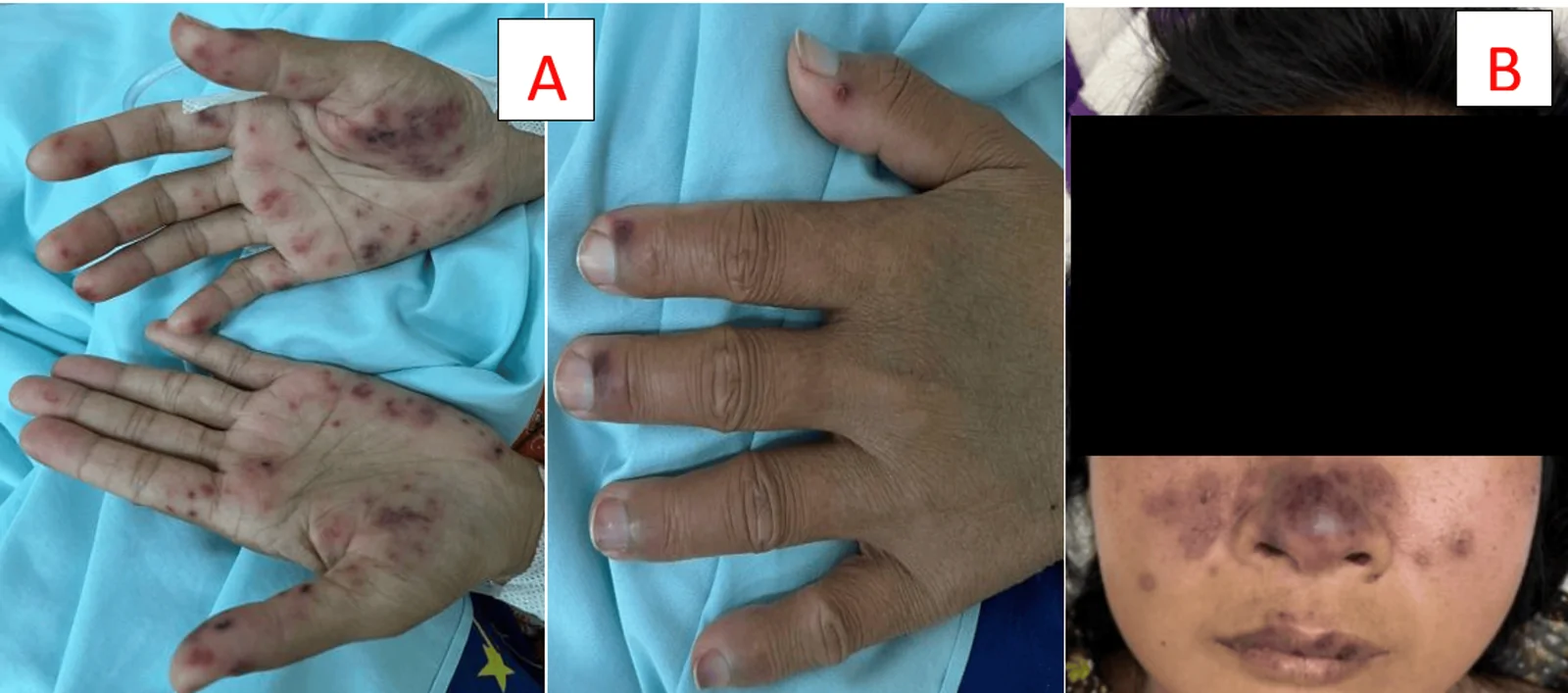
Cutaneous lupus erythematosus with palmar erythema (A) and malar rash (B).

Joint examination showed mild swelling and tenderness of the wrists and knees without effusion. Cardiovascular examination was unremarkable with no murmurs and no peripheral edema. Respiratory and neurological examinations were normal. Laboratory examination revealed pancytopenia, as well as elevated transaminases and troponin levels (Table 1).

**Table 1:** Clinical laboratory results. MCV: mean corpuscular volume; MCH: mean corpuscular hemoglobin; MCHC: mean corpuscular hemoglobin concentration; INR: international normalized ratio; PTT: prothrombin time; CRP: C-reactive protein; BUN: blood urea nitrogen; eGFR: estimated glomerular filtration rate; AST: aspartate aminotransferase; ALT: alanine aminotransferase; TIBC: total iron-binding capacity

Parameter	Result	Reference range (adult)
White blood cell, ×10⁹/L	1.47	4.5–11.0
Hemoglobin, g/L	88	120–160 (female) 140–170 (male)
Platelet Count, ×10⁹/L	31	150–450
MCV, fL	72.7	80–100
MCH, pg	23.3	27–31
MCHC, g/dL	32.1	32–36
INR	1.5	0.8–1.2
CRP, mg/L	17.56	<5.0
Procalcitonin, ng/mL	0.574	<0.15
Glycemia, g/L	0.84	0.70–1.00
BUN, mg/dL	30	7–20
Serum creatinine, µmol/L	195	60–100
eGFR, mL/minute/1.73m^2^	25	90–120
Albuminemia, g/L	29	35–55
Proteinuria, mg/24 hours	600	<150
Lactic acid, mmol/L	2.31	0.5–2.2
Troponin, ng/mL (high sensitivity)	2.13	<0.04
Sodium, mmol/L	130	135–145
Potassium, mmol/L	4.6	3.5–5.0
Chloride, mmol/L	100	95–105
Alkaline phosphatase, U/L	138	30–120
AST, U/L	351	10–40
ALT, U/L	245	7–56
Serum iron, µmol/L	4.42	11–30
TIBC, µmol/L	42	45–70

The serial troponin was performed showing mild elevation. The thyroid function was normal, and the blood culture was negative. Serology for HIV and hepatitis B and C was negative. Urinalysis showed proteinuria (600 mg/24 hours). Immunological testing showed a positive anti‑nuclear antibody (ANA) at a titer of 1:2,560 (homogeneous pattern) with positive anti‑Ro/SSA and anti‑RNP/sm antibodies. Anti‑La/SSB, anti‑double‑stranded DNA, and anti‑cardiolipin antibodies were negative (Table 2).

**Table 2:** Antinuclear antibody profile (16 autoantibodies). nRNP/Sm: ribonucleoprotein/Smith; SS-A: Sjögren’s syndrome-related antigen A; Ro-52: a specific 52 kDa protein within the Ro/SS-A antigenic complex; SS-B: Sjögren’s syndrome-related antigen B; Scl-70: scleroderma-70; PM-Scl100: polymyositis-scleroderma 100; Jo-1: histidyl-tRNA synthetase; CENP B: centromere protein B; PCNA: proliferating cell nuclear antigen; dsDNA: double-stranded deoxyribonucleic acid; Rib. P-Prot: ribosomal P-proteins; AMA M2: anti-mitochondrial antibody M2 subtype; DFS70: dense fine speckled 70 kDa protein

Test name	Result
nRNP/Sm	Positive
SS-A	Positive
Ro-52	Negative
SS-B	Negative
Sci-70	Negative
PM-Scl100	Negative
Jo-1	Negative
CENP B	Negative
PCNA	Negative
dsDNA	Negative
Nucleosomes	Negative
Histones	Negative
Rib.P-Prot	Negative
AMA M2	Negative
DFS70	Negative
Method	Immunoblot

The electrocardiogram (ECG) showed CHB (third‑degree) with a junctional escape rhythm at 46 beats/minute, narrow QRS complexes (90 ms) with negative T waves from V1 to V4 (Figure 2).

**Figure 2:**
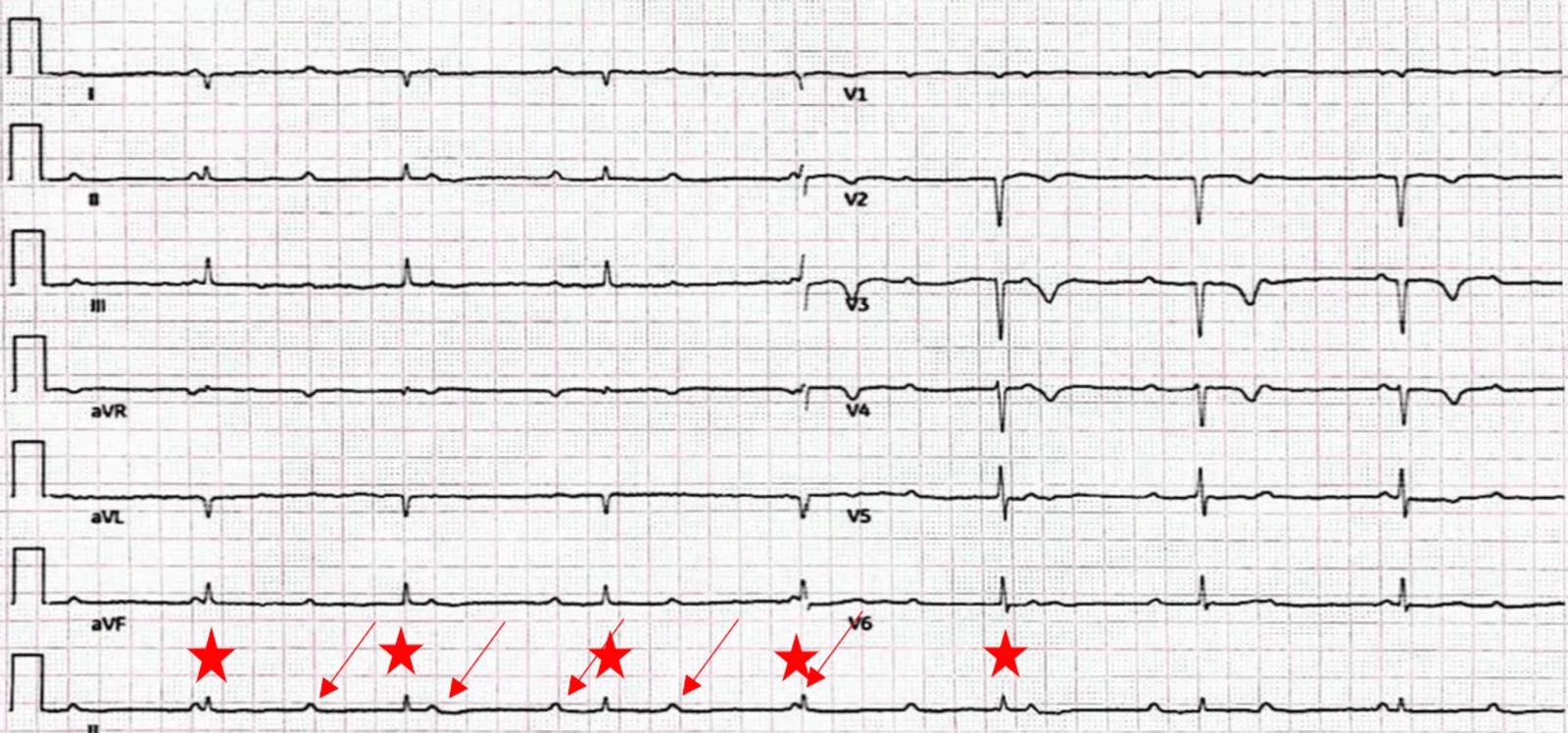
Electrocardiogram. The electrocardiogram showed atrioventricular dissociation (red arrow) with a junctional escape rhythm at 46 beats/minute with a narrow QRS complex (red asterisk) and negative T waves from lead V1 to V4.

A transthoracic echocardiogram showed minimal pericardial effusion with preserved left ventricular ejection fraction and no valvular abnormalities (Figure 3).

**Figure 3:**
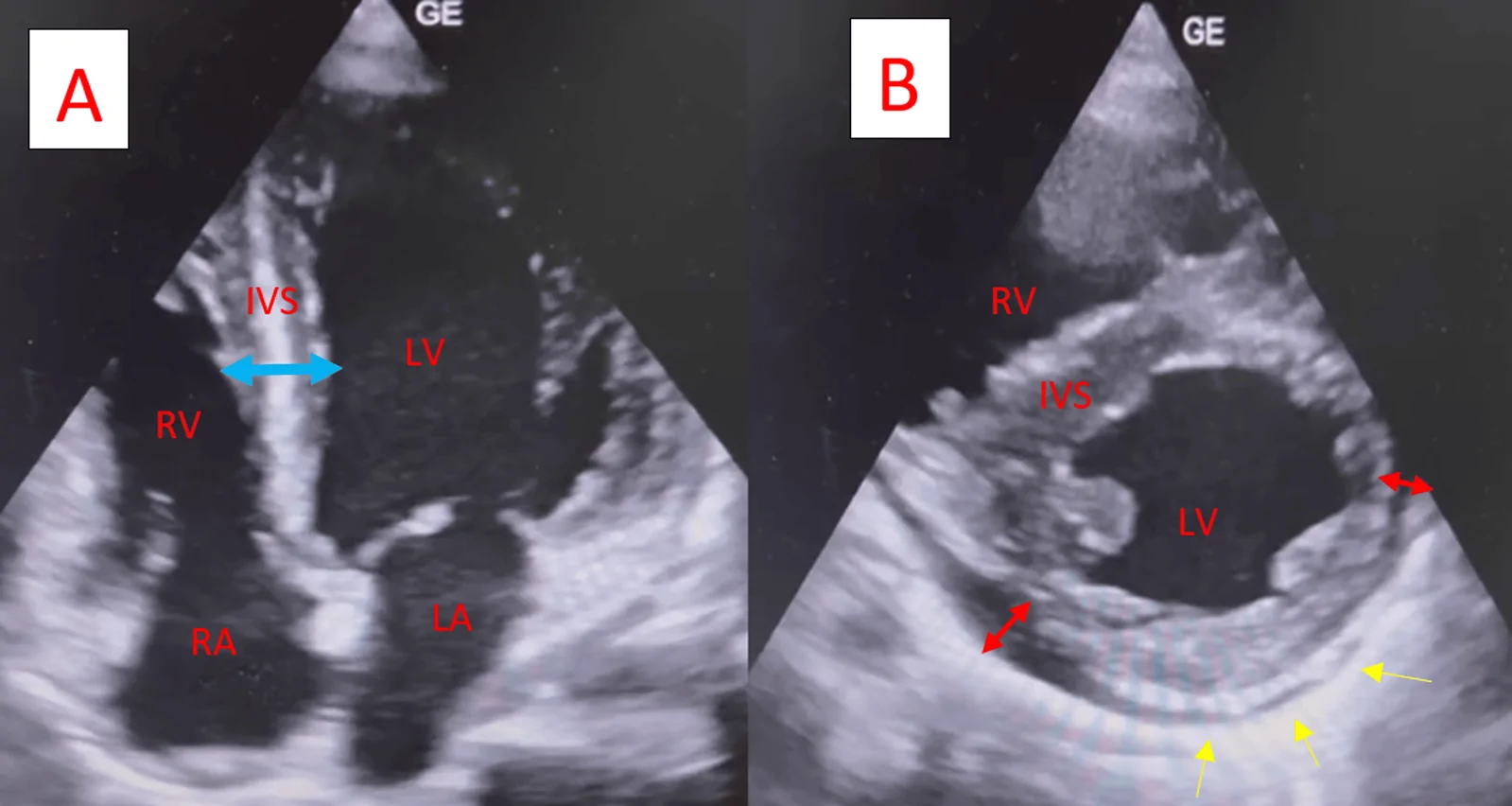
Transthoracic echocardiography. Panel A: Concentric wall thickening of LV in apical four-chamber view (end-diastolic diameter of interventricular septum is 13 mm) (blue arrow) with preserved LV ejection fraction. Panel B: Minimal pericardial effusion in parasternal short-axis view (B) (end-diastolic diameter is 9 mm) (red arrow) and focal echobright at the posterior wall of LV (yellow arrow) with discrete hypokinesia. RV: right ventricle; LV: left ventricle; RA: right atrium; LA: left atrium; IVS: interventricular septum

The coronary angiography was normal without stenosis. The 24-hour Holter monitor revealed persistent complete AV block with a ventricular rate that varied between 46 and 55 beats/minute without any ventricular pauses longer than 3 seconds or evidence of ventricular tachyarrhythmia. Due to limited resources, cardiac magnetic resonance imaging is currently unavailable at our center.

Following a multidisciplinary discussion among our electrophysiologist, rheumatologist, and nephrologist, the patient was diagnosed with CHB secondary to myopericarditis in the context of SLE, based on clinical, paraclinical, and echocardiographic findings. The SLE management plan consists of a three-day consecutive course of intravenous methylprednisolone (15 mg/kg of body weight) transitioning to oral prednisolone for three to six months before tapering, daily hydroxychloroquine (Plaquenil) (400 mg per day), and a stepped mycophenolate mofetil regimen starting at a lower daily dose for one week (1 g per day for one week and then increase to 2 g per day if no side effect), increasing to a higher dose for three to six months (3 g per day), and eventually maintaining at a lower dose for one to two years (1 g per day) based on clinical response [2]. Despite a daytime heart rate of 46-55 beats per minute, an asymptomatic presentation, and no signs of hemodynamic compromise, a temporary transvenous pacemaker was inserted according to guidelines to prevent sudden cardiac death [3]. ECG monitoring was continued, and the patient was kept at bed rest with continuous telemetry.

After 10 days of glucocorticoid and immunosuppressive therapy, the complete AV block resolved to a first‑degree AV block (PR interval 240 ms) with a sinus rate of 70 beats/minute. The patient’s fever, rash, and arthralgias also improved markedly. She was discharged on a tapering regimen of prednisolone, mycophenolate mofetil, and hydroxychloroquine. At three‑month follow‑up, the ECG remained stable with first‑degree AV block, and the patient had no cardiac symptoms.

## Discussion

Based on the clinical presentation, electrocardiographic findings, and laboratory results, this patient was diagnosed with SLE with cardiac, cutaneous, hematological, and renal involvement [4]. The pathophysiology of heart block in SLE is likely multifactorial. The most widely accepted mechanism involves an autoimmune-mediated injury to the cardiac conduction system. Pathological studies have shown lymphocytic infiltration and fibrosis in the conduction tissue of SLE patients with heart block. The presence of anti-Ro/SSA and anti-La/SSB antibodies, which are frequently found in SLE and Sjögren’s syndrome, has been implicated, but a direct causal relationship between these antibodies and acquired complete AV block in adults remains uncertain. These autoantibodies can cross-react with fetal and adult cardiac tissue, potentially binding to and inhibiting L-type and T-type calcium channels on cardiomyocytes, leading to conduction slowing and heart block. This patient was known to be positive for anti-Ro/SSA antibodies, supporting this pathophysiologic link. It has been proposed that the same antibody-mediated process responsible for congenital heart block in neonatal lupus could also be a factor in acquired CHB in adults. While some studies have found an association between CHB and anti-ENA antibodies, other reviews have failed to confirm a consistent relationship, with some reporting a trend toward HLA-DR3 association [5-7]. Other potential etiologies include direct inflammatory involvement of the myocardium (myocarditis) or the conduction system itself, or, rarely, cardiotoxicity from medications such as hydroxychloroquine, especially with long-term, high-dose use [8].

A review of 31 published cases of CHB in SLE found that syncope was the most common presenting symptom, and asymptomatic conduction disease was not a typical presentation, with CHB occurring almost exclusively in females at a median age of 37 years [9]. We report a case of newly diagnosed SLE presenting with fever and butterfly rash in which ECG unexpectedly revealed asymptomatic complete AV block. This case has several noteworthy features. First, it confirms that high‑grade AV block can occur as a completely silent finding in active SLE, even at disease onset. Asymptomatic CHB is rarely described, and to our knowledge, this is one of the few reports in a non‑pregnant adult. Second, the complete AV block resolved completely with immunosuppressive therapy alone, without requiring pacemaker implantation. This reversibility is not always observed; in many reports of SLE‑associated complete heart block, the conduction abnormality is permanent and necessitates a permanent pacemaker [10]. The reversibility of the block in some patients, as in our case, suggests that early inflammation may be more amenable to immunosuppression before irreversible scarring develops. Cardiac magnetic resonance imaging (MRI) with late gadolinium enhancement can help detect myocardial or conduction system inflammation, but unfortunately, Cardiac MRI was not performed as the technology is unavailable due to resource limitations.

The management of asymptomatic CHB in the context of active SLE is complex. In some reported cases, CHB resolves with the treatment of an acute lupus flare or following the withdrawal of an offending agent such as an antimalarial drug, often in conjunction with corticosteroid therapy.

## Conclusions

This case of CHB in a young patient with multi-organ SLE is a critical reminder that serious conduction abnormalities can occur without typical symptoms, and this rare presentation underscores the need for cardiac evaluation, including a baseline ECG. A proactive, multidisciplinary approach involving rheumatology and cardiology is essential for early detection and for mitigating the risk of complications such as sudden cardiac death. Future prospective studies are needed to better delineate subgroups of lupus patients who might achieve sustained conduction recovery through medical therapy alone versus those requiring immediate, lifelong pacing.
